# How integrated are behavioral and endocrine stress response traits? A repeated measures approach to testing the stress-coping style model

**DOI:** 10.1002/ece3.1395

**Published:** 2015-01-11

**Authors:** Kay Boulton, Elsa Couto, Andrew J Grimmer, Ryan L Earley, Adelino V M Canario, Alastair J Wilson, Craig A Walling

**Affiliations:** 1 Institute of Evolutionary Biology, University of EdinburghWest Mains Road, Edinburgh, EH9 3JT, U.K; 2 CCMar, University of AlgarveCampus de Gambelas, 8005-139, Faro, Portugal; 3 College of Life and Environmental Sciences, University of ExeterCornwall Campus, Treliever Road, Penryn, Cornwall, TR10 9EZ, U.K; 4 Department of Biological Sciences, University of Alabama300 Hackberry Lane, Box 870344, SEC Building, Tuscaloosa, Alabama, 35487

**Keywords:** 11-ketotestosterone, Boldness, cortisol, multivariate behavior, repeatability, waterborne steroid collection

## Abstract

It is widely expected that physiological and behavioral stress responses will be integrated within divergent stress-coping styles (SCS) and that these may represent opposite ends of a continuously varying reactive–proactive axis. If such a model is valid, then stress response traits should be repeatable and physiological and behavioral responses should also change in an integrated manner along a major axis of among-individual variation. While there is some evidence of association between endocrine and behavioral stress response traits, few studies incorporate repeated observations of both. To test this model, we use a multivariate, repeated measures approach in a captive-bred population of *Xiphophorus birchmanni*. We quantify among-individual variation in behavioral stress response to an open field trial (OFT) with simulated predator attack (SPA) and measure waterborne steroid hormone levels (cortisol, 11-ketotestosterone) before and after exposure. Under the mild stress stimulus (OFT), (multivariate) behavioral variation among individuals was consistent with a strong axis of personality (shy–bold) or coping style (reactive–proactive) variation. However, behavioral responses to a moderate stressor (SPA) were less repeatable, and robust statistical support for repeatable endocrine state over the full sampling period was limited to 11-ketotestosterone. Although *post hoc* analysis suggested cortisol expression was repeatable over short time periods, qualitative relationships between behavior and glucocorticoid levels were counter to our *a priori* expectations. Thus, while our results clearly show among-individual differences in behavioral and endocrine traits associated with stress response, the correlation structure between these is not consistent with a simple proactive–reactive axis of integrated stress-coping style. Additionally, the low repeatability of cortisol suggests caution is warranted if single observations (or indeed repeat measures over short sampling periods) of glucocorticoid traits are used in ecological or evolutionary studies focussed at the individual level.

## Introduction

When challenged by adverse and uncontrollable environmental stimuli, animals use behavioral and physiological components of the stress response to maintain homeostasis (Selye 1973; Johnson et al. 1992; Chrousos 1998) and minimize loss of fitness (Levine and Ursine 1991; Blas et al. 2007; Breuner et al. 2008; Koolhaas et al. 2011). Stress response may vary among individuals within a population (Huntingford 1976; Verbeek et al. 1996; Devries 2002), a phenomenon that has led researchers to postulate the existence of “stress-coping styles” (SCS) (Benus et al. 1991; Koolhaas et al. 1997, 1999; Korte et al. 2005). Under the SCS model, it is widely expected that behavior and physiology will be integrated within divergent coping styles typically characterized as being either proactive or reactive (Koolhaas et al. 1997). Proactive individuals actively challenge stressors and present behavioral profiles consistent with bold personalities (e.g., Brown et al. 2007; Thomson et al. 2011), rapidly develop rigid routines and usually have low hypothalamic–pituitary–adrenal (HPA) (or in fishes hypothalamic–pituitary–interrenal (HPI) activity). In contrast, reactive individuals demonstrate low levels of aggression and appear to be more flexible in their behavioral responses, tending toward raised HPA/HPI activity (e.g., Øverli et al. 2007; Carere et al. 2010). Although often presented as dichotomous, proactive and reactive coping styles may actually represent opposite ends of a continuously varying axis of SCS (Barreto and Volpato 2011). If the SCS model is valid, then stress response traits should not only be repeatable, but physiological and behavioral responses ought to change in an integrated manner along a major axis of among-individual variation, that is, there should be strong among-individual covariation between physiological and behavioral responses (Wechsler 1995). Here, using a freshwater fish population, we investigate among-individual (co)variation in behavioral and endocrine stress response traits to test these predictions and thus evaluate the SCS.

In general, studies of vertebrate stress responses have focused primarily on neuroendocrine physiology. Much is now known about the general mechanisms whereby stress exposure stimulates uptake and transfer of oxygen, reallocates energy away from reproduction and growth and, under chronic exposure, suppresses immune function (Wendelaar Bonga 1997). Despite this, comparatively few studies to date have directly tested for repeatable, among-individual variance in stress-related endocrine traits (but see e.g., Andrade et al. 2001; Ferrari et al. 2013). Nonetheless, genetic studies have provided evidence of heritable variation for endocrine response to stress in many taxa (e.g., Silberg et al. 1999; Evans et al. 2007), and a trait cannot be heritable without being repeatable. In fishes, genetic variation for plasma cortisol (F) levels has been demonstrated widely (e.g., Pickering and Pottinger 1989; Fevolden et al. 1993; Barton 2002; Pottinger 2010). Artificial selection on rainbow trout (*Oncorhynchus mykiss*) has successfully generated high and low poststress cortisol lines (Pottinger and Carrick 1999), while quantitative trait loci (QTL) for endocrine stress response traits have been mapped in several aquaculture species (Massault et al. 2010; Boulton et al. 2011).

Even though endocrine processes may be important for coping with acute stress challenges, it should also be recognized that behavioral responses such as freezing, fighting, or fleeing may be critical in some contexts (e.g., response to predation attempt) (Blanchard et al. 1998). There is evidence for alternative behavioral stress response profiles in rodents (Benus et al. 1991; Sgoifo et al. 1998; Koolhaas et al. 1999; Veenema 2009), birds (e.g., Carere et al. 2003; Fraisse and Cockrem 2006), and livestock (Hessing et al. 1994). In many cases, associations between single behaviors and HPA activity have been found, consistent with SCS (e.g., Sutherland and Huddart 2012; Wesley et al. 2012). More generally, empirical studies in the burgeoning field of animal personality (Sih et al. 2004; Réale et al. 2007) have emphasized that among-individual (i.e., repeatable) variation in behavior is taxonomically widespread. This is certainly true for behaviors associated with stress exposure (e.g., Wilson 1998; Gosling and John 1999; Briffa et al. 2008; Rudin and Briffa 2012), leading some authors to argue that SCS and personality are closely related concepts (at least as applied to animals) if not necessarily synonymous (Connor-Smith and Flachsbart 2007; Øverli et al. 2007; Castanheira et al. 2013).

Along a reactive–proactive axis of SCS, behavior is expected to change in a manner broadly corresponding to the axis of “shyness–boldness” described in the personality literature (Wilson et al. 1994; Winberg et al. 2007; for example, Budaev 1997; Huntingford et al. 2010; Raoult et al. 2012). Empirical studies demonstrating variation in boldness have been conducted in many taxa including fishes (e.g., Budaev et al. 1999; Bell et al. 2009). While there is some evidence of association between endocrine and behavioral stress response traits in a range of taxa (e.g., Andrade et al. 2001; Creel 2001; Thaker et al. 2009; Archard et al. 2012), few studies have incorporated repeated observations on both traits (but see Ellis et al. 2004; Sebire et al. 2007; Ferrari et al. 2013). This is an important limitation because repeated measures are required to partition the among-individual differences expected under the SCS model from sources of within-individual (i.e., observation specific) variation (Dingemanse et al. 2010; Dochtermann and Roff 2010; Dingemanse and Dochtermann 2013). Therefore, two key questions remain largely unanswered. Firstly, to what extent are endocrine stress responses a repeatable phenotype of the individual? Secondly, assuming that correlations between behavioral and endocrine stress responses are apparent, to what extent are these driven by among-individual (repeatable) differences, and do they mirror patterns expected under SCS?

Here, we aim to address these questions using a small tropical freshwater fish, *Xiphophorus birchmanni*. In this species, we have previously demonstrated a strong axis of among-individual variation in boldness that is stable over long periods, that is, representative of expected life span (Boulton et al. 2014). We now expand on this previous work to ask whether there is also among-individual variation in endocrine physiology, and whether behavioral and endocrine responses to a stressor are integrated in a manner consistent with SCS. To investigate behavioral response, we subject fish to a modified open field trial (OFT, a mildly stressful novel situation), coupled with a simulated predator attack. We used a modified decoy heron for this purpose as members of the Ardeidae family are known to predate the Arroyo Coacuilco river (near Coacuilco, municipality of San Felipe Orizatlán, Hidalgo, Mexico) where the population of fish studied was ancestrally sourced (GG Rosenthal, personal communication). To investigate endocrine state, we quantify cortisol (F), the principal, and most frequently measured glucocorticoid in fishes released by activation of the hypothalamic–pituitary–interrenal (HPI) axis on exposure to stressors (Mommsen et al. 1999). In addition, we quantify 11-ketotestosterone (11KT), an important androgen in teleosts (Mayer et al. 1990; Mommsen et al. 1999). Although not normally considered a stress hormone *per se*, many studies point toward a link between gonadal steroids and personality traits such as aggression and boldness (Pellis and Mckenna 1992; Borg and Mayer 1995; Oliveira et al. 2002; Taves et al. 2009; Koolhaas et al. 2010). Here, we seek to test three specific predictions: (1) that fish exposed to stressors differ consistently in behavioral responses thus aligning with expectations under a shy–bold personality paradigm; (2) that there is repeatable variation for prestressor endocrine state and/or change in hormone levels following stress exposure; (3) that behavioral and endocrine stress response traits (co)vary and correlation exists at the among-individual level, with bolder individuals having lower HPA/HPI activity as predicted by the SCS model.

## Methods

### Animal husbandry

**Figure 1:**
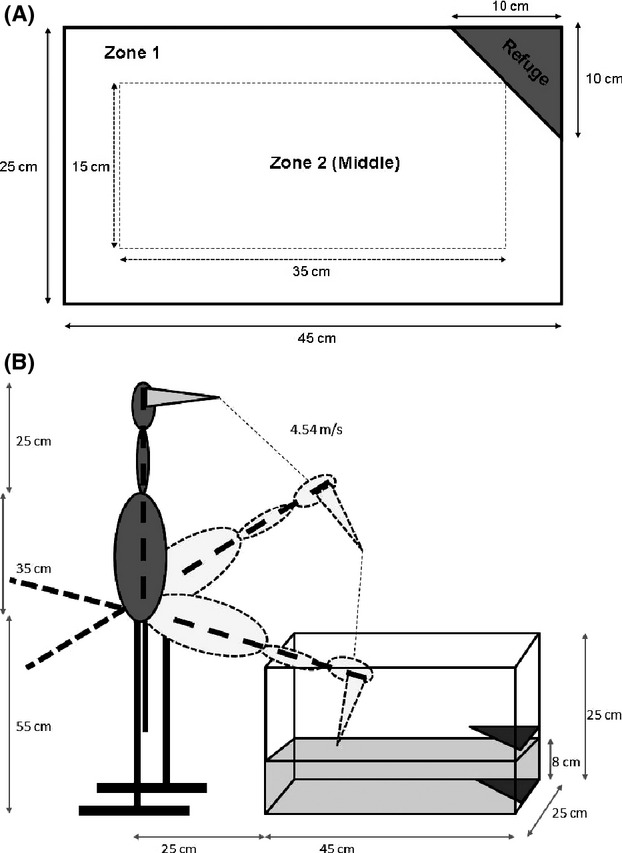
Setup of experimental arena for behavioral trials showing (A) an overhead view with tank dimensions, and (B) the position and dimensions of the decoy heron used to simulate an avian predation event. Zones 1 and 2 are defined for scoring by tracking software only and were of equal area. The refuge comprises a triangle of aquarium filter foam taped just above the water level (2.5 cm) to give the impression of a bank to hide under. A piece of card (of similar size and shape) was also placed under this corner of the tank. The decoy heron was positioned so as not to cast a shadow over the arena, its downward swing constrained to stop with the beak at water level.

Twenty male *Xiphophorus birchmanni* (Fig.1) were sampled haphazardly from a stock tank containing second-generation captive-bred fish. Animals were of unknown age but of similar size (1.16 ± 0.073 g) and developmental stage. All were sexually mature based on external assessment of gonopodium development. Fish were then housed individually in half sections of ten 30 L (37 × 37 × 22 cm) tanks, divided by opaque, water permeable dividers constructed from Perspex frames covered with dark-colored fine-gauge nylon net. Ten half-tanks were contained within a stack sharing a common recirculating water supply; thus, within a stack fish were physically and visually, although not chemically isolated. Individual rather than group housing was used to prevent among-individual variation in exposure to social stress caused by dominance interactions (i.e., subordinates being subject to higher aggression) that are well characterized in male swordtails (Earley 2006; Wilson et al. 2013). Fish were maintained at 21–23°C on a 12:12 light:dark cycle. Fish were fed twice per day, using a mix comprising equal quantities of crushed spirulina (ZM systems, U.K.: http://www.zmsystems.co.uk/) and brine shrimp flake in the morning followed by a previously frozen mixture of bloodworm, brine shrimp nauplii, and daphnia in the late afternoon. Fish were not fed on the morning of days when they underwent trials.

### Behavioral trials

Following collection of a pretrial water sample for hormone assay (see below), each fish was placed in an empty 45 × 25 × 25 cm glass tank filled to a depth of 8 cm with 9 L of clean water. The tank was positioned on an illuminated light box (illuminated area of 594 × 420 mm with an LED light source of approximately 1500 lux and color temperature of 9000 kelvin), increasing contrast to allow data extraction using video-based tracking software. A small refuge was created in the tank by attaching a triangular piece of aquarium filter foam (10 × 10 × 14 cm) just above the water (2.5 cm) in one corner (Fig.1A). An equally sized piece of card was placed below the tank in the same corner. Thus when within the refuge, the fish was not visible from above and was shielded from light coming from below. A Sunkwang C160 video camera fitted with a 5–50 mm manual focus lens was suspended above the apparatus.

Following introduction to the tank, each fish was allowed 300 sec to acclimate to the experimental arena and thereafter behavior was recorded for 120 sec (at 15 frames s^−1^) on video (described below). Note that being placed in a novel environment is considered to be a mild stress stimulus in small fishes (Burns 2008). A further (moderate) acute stress exposure was then imposed, using a plastic decoy heron fixed to a home-made swinging stand to simulate an avian predation event (Barber et al. 2004) (Fig.1B). The decoy was positioned in such a way that it did not create a shadow over the arena in the upright position. When released, the decoy swung down rapidly (accelerating to approximately 4.5 m s^−1^) toward the tank. The swing was limited to stop the decoy abruptly (with the beak at water level) causing a loud percussive sound and vibration that disturbed the tank. A further 120 sec of behavior was recorded before the fish was removed for collection of the post-trial water sample. Water in the experimental tank was replaced prior to the next trial. The entire sampling process was repeated five times at 4 day intervals. All fish were sampled on each occasion (in variable order, to avoid confounding any diurnal effects with individual identity) with the exception of one individual that died between the fourth and fifth trials. Two 165 L glass tanks (122 × 45 × 30 cm) were used to store water at room temperature to supply the behavior trials and hormone collection beakers (see below).

Data were extracted from videos using tracking software from Biobserve (http://www.biobserve.com/products/viewer/index.html). Specifically, for the 120-sec period before the heron strike, we measured track length (TL, total distance moved in cm); percentage of time being active (ACT; defined as moving at >1.5 cm s^−1^); percentage of tank basal area covered (AC); time in middle of tank (TIM, in sec, Fig.1A). These traits were selected based on a previous independent study using a slightly different experimental arena (the same tank but with no refuge) that showed them to be repeatable and indicative of a major axis of boldness variation in this population of *X. birchmanni* (Boulton et al. 2014). In addition, we recorded time spent out of the refuge (TOR), our *a priori* expectation being that this would be consistently higher in bold individuals. Based on pilot data, we had expected all fish to respond to the acute stressor (simulated predation event) by immediately entering the refuge and indeed this was observed in all but two trials. However, while we had planned to use a continuous measure of latency to re-emerge as a further metric of behavioral stress response, in approximately two-thirds of trials, the fish did not re-emerge within the subsequent two-minute observation period. Due to this data censoring, we used emergence from the refuge (emREF) as a binary behavioral response to the acute stressor (1 the fish re-emerged, 0 it did not). Although continuously varying emergence times could have been collected for more (or all) fish by extending the postpredation observation period (or observing until emergence), this would have negatively impacted our ability to manage the endocrine sampling (conducted immediately before and after trials; see below) without compromising sample size.

### Endocrine assays

We used a noninvasive method to assess individual endocrine state from holding water samples (Ellis et al. 2004). This allows repeated sampling of small fish that would not survive invasive collection of blood plasma for assay. Water samples were collected pre- and postbehavioral trial as follows. Non-PET plastic inserts for 500-mL glass beakers were custom-made by cutting the neck from cylindrical 500-mL opaque Nalgene bottles and drilling drainage-holes into the base (following Archard and Braithwaite 2011). These inserts were used to capture and transfer fish from tanks to beakers on all occasions. First, fish in home tanks were quickly (typically <5 sec) captured with the insert, then immediately and gently lifted from the tank (allowing water to drain) before being placed in a glass beaker containing 500 mL clean water. Capture and handling time, that is, transfer to beaker of clean water, was not recorded, but took no longer than 60 sec in any given case. The beaker was covered with a dark net and left for 60 min to obtain the pretrial endocrine sample. The insert was then used to transfer the fish to the behavioral trial arena tank by raising it from the beaker and then immersing it in the tank; this was all performed in such a way as to minimize the disturbance experience by the fish. After the behavioral trial, a clean insert was used to quickly catch the fish and transfer to a second beaker of 500 mL water for a further 60-min period to collect the post-trial endocrine sample. Fish were then removed from the beaker using the insert and placed onto a dry paper towel positioned on digital scales, where they were weighed (to the nearest 0.01 g) before being returned to home tanks. Nitrile gloves were worn throughout all procedures requiring contact with fish or holding water. After use, all beakers and inserts were rinsed thoroughly with distilled water then ethanol and allowed to dry overnight.

Each 500 mL water sample was filtered to remove any debris (Whatman Filter paper, grade 1, 24 cm) and steroids were extracted to C18 solid phase columns (SepPak® Vac 3 cc/500 mg; Waters Inc., Milford, MA) previously primed (2 × 2 mL HPLC-grade methanol followed by 2 × 2 mL distilled water). Solid phase extraction was conducted under vacuum pressure using a twenty-port manifold (waters, as before) and Tygon tubing (Saint Gobain, Formulation 2275) to transfer samples from beaker to column. Columns were stored at −20°C until the end of the behavioral data collection, when all columns were packed in dry ice and despatched to CCMar, Universidade do Algarve, Faro, Portugal, for quantification of waterborne hormone levels by radio-immunoassay (RIA). Columns were defrosted at 4°C and activated by washing with 2 × 2 mL deionized water to purge any salts. Steroids were eluted into glass tubes with ethanol (3 × 1 mL). The ethanol was evaporated at 42°C under nitrogen gas and the residue resuspended in 1 mL RIA buffer (gelatine phosphate 0.05 mol/L, pH 7.6).

RIA was used to quantify levels of free F and 11KT. For the cortisol RIA, we used an antiserum raised in rabbit against cortisol-3-CMO-BSA (ref 20-CR50; Fitzgerald Industries International, Concord, MA). Cross-reactivities were 54% for 11-desoxycortisol, 10% for cortisone, 16% for 17,21-dihydroxy-5*β*-pregnan-3,11,20-trione, 5% for 11*β*,17,21-trihydroxy-5*β*-pregnan-3,20-dione, 0.05% for 11*β*-hydroxytestosterone and less than 0.001% for testosterone. The 11-ketotestosterone antiserum cross-reactivities are given elsewhere (Kime and Manning 1982). To verify the specificity of the RIAs toward the samples, a pool of water extracts was first separated by normal phase thin-layer chromatography and fractions assayed for the two steroids. The two RIAs were shown to be highly specific, only cross-reacting with single fraction comigrating, respectively, with F and 11KT. Inter- and intra-assay variability for the two assays was below 12%.

### Validation of waterborne steroid assays

That waterborne steroid assays may predict plasma and/or whole-body concentration has been demonstrated in a number of fish species (e.g., Scott and Liley 1994; Ellis et al. 2007; Sebire et al. 2007). However, the method has not previously been used in *Xiphophorus birchmanni,* and we therefore tested the relationship between steroid concentrations in water and whole fish. Twenty-six randomly selected stock fish of mixed sex, age, and size were held separately in 500-mL glass beakers for 60 min as described above. They were then immediately euthanized by transfer to a beaker containing an MS22 solution (50 g/L) buffered with an equal quantity of sodium bicarbonate. Fish were weighed (to the nearest 0.01 g), then frozen whole at −20°C before being shipped to CCMar. Waterborne samples were processed as described above. Whole fish samples were individually pulverized in liquid nitrogen with a mortar, transferred to glass extraction tubes, mixed with 5-mL absolute ethanol (Merck 1.00983.5000), vortexed for 10 min and centrifuged. The supernatant was aspirated to a second extraction tube, evaporated, and resuspended in 200 *μ*L distilled water. Free steroids were extracted twice with 3 mL diethyl ether (VWR 23811.292), the solvent dried with nitrogen gas, and the extracts resuspended in radioimmunoassay buffer. Steroid release rates (pg/h) determined from pre- and post-trial collections and sacrificed fish were natural-log (Ln)-transformed for analysis.

### Statistical analysis

Data were analyzed using (multivariate) linear mixed effect models parameterized by restricted maximum likelihood with the statistical package, ASReml V3, (Gilmour et al. 2009). As this software does not readily accommodate non-Gaussian traits, we analyzed the binary behavioral response trait emREF using a Bayesian approach implemented in MCMCglmm (Hadfield 2010a). In all models, the inclusion of fish identity as a random effect allowed the observed phenotypic (co)variance structure to be partitioned into among-individual (**I**) and within-individual (residual, **R**) between-trial components (note bold font is used here to denote matrices). Prior to analysis, data were square root (all behaviors except emREF) or natural-log-transformed (endocrine traits) to meet assumptions of normality. After transformation, all data were rescaled to standard deviation units. This rescaling was carried out for two reasons: firstly, it simplifies the interpretation of results as the estimated among-individual variance (*V*_**I**_) for any (transformed) trait corresponds to the repeatability (defined as the proportion of observed phenotypic variance explained by individual identity); secondly, for the inference of a latent personality trait, this prevents any single observed behavior from dominating **I** due to scaling effects alone (Wilson et al. 2013). For all traits, we fitted fixed effects of *mean*,*trial number* (the cumulative number of trials experienced by an individual), home *stack* (a two level factor accounting for sets of fish sharing the same water supply), and *day order* (modeled as a linear effect of the number of preceding trials performed that day). *Day order* provides a statistical control for any diurnal patterns in average response variables, while *trial number* was included to control for the possibility of trait means changing across repeated trials (e.g., as a consequence of habituation and/or learning). For endocrine traits, we also included *mass* as an additional fixed effect. This allowed us to account for the expected increase in hormone release rate with size due to diffusion into the holding water across a larger gill area (Ellis et al. 2004). The covariates *day order* and *mass* were both mean-centered. For models fitted using REML, the significance of fixed effects was tested by Wald *F*-tests, while likelihood ratio tests (LRT) were used to assess the significance of the random effect of fish identity. For models fitted using MCMCglmm, statistical inference was based on the posterior distributions of estimated parameters.

### Estimating behavioral coping style

First, we modeled the set of baseline behavioral traits observed prior to the simulated predation event. This was to test our *a priori* expectation that there would be among-individual variance and covariance structure consistent with the presence of an axis of boldness variation. We initially fitted a multivariate model with no random effects, such that all variance was allocated to the residual (within-individual) component **R**, specified as a “diagonal” matrix (model 1) where trait variances are estimated but all among-trait covariance terms are set to equal zero. This model was compared to a second model (model 2), where fish identity was fitted as a random effect, and the among-individual component **I** was specified as a second diagonal matrix structure. This allowed a global test (i.e., across all baseline behavior traits) of among-individual variance by comparing models 1 and 2 with a likelihood ratio test (LRT) following Wilson et al. (2010). For comparing multivariate models in this way, we conservatively assume that twice the difference in model log-likelihoods is distributed as 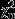
_**,**_ where the DF (*n*) is equal to the additional number of parameters to be estimated in the more complex model, in this case five. Note that for univariate model comparisons as presented in supporting materials, we modify the test following recommendations presented by Stram and Lee (1994) and Visscher (2006). We then modeled between-trait covariance in **R** (within-individual, model 3) and in both **I** and **R** (among- and within-individuals, model 4), allowing us to test whether behaviors covary (model 3 vs. 2) and whether among-individual differences contribute significantly to this covariance (model 4 vs. 3). In model 4, **I** is therefore estimated as a fully unstructured matrix (i.e., both variances and covariances allowed), with trait-specific variance (*V*_I_) estimates on the diagonal (equal to the trait repeatabilities) and the among-individual covariance (COV_Ix,y_) between each pair of traits (*x*,*y*) off the diagonal. Among-individual correlations (*r*_**I**_) were then calculated by rescaling the among-individual covariance (COV_I(*x*,*y*)_) so that *r*_*x*,*y*_ = COV_I(*x*,*y*)_/√(*V*_Ix_*V_Iy_).

Eigenvector (EV) decomposition was then used to evaluate whether **I** among this set of traits (as estimated under model 4) was dominated by a single major axis interpretable as boldness. Specifically, based on previous findings in an independent data set, we predicted that the first eigenvector of **I** (EV1_I_) would capture most of the among-individual behavioral variance and would be characterized by trait-specific loadings of equal sign and similar magnitude. We used parametric bootstrapping (Boulton et al. 2014) to simulate 5000 replicate draws of **I** from a multivariate normal distribution with means and variances defined by the REML estimate of **I** and its sampling variance–covariance matrix, respectively. Each matrix was then subjected to eigen analysis, and we used the 95% highest probability density (HPD) interval of parameter distributions to describe uncertainty around the trait loadings on EV1_I_.

We then estimated the repeatability of emREF (univariate model) and its among-individual correlations with the baseline behaviors observed prior to the predator strike using bivariate models in MCMCglmm (Hadfield 2010a,b). Emergence was treated as a categorical trait with residual variance fixed at 1. All (transformed) open field trial (OFT) traits were treated as Gaussian. MCMCglmm models were run for 1,050,000 iterations with a burnin of 50,000 iterations and a thinning interval of 1000 iterations. The repeatability of emREF on the liability scale was determined as the intraclass correlation, calculated as *V*_I_/(*V*_I_ + *V*_R_ + *π*^2/3^), where *V*_I_ is the among-individual variance and *V*_R_ is the residual variance (i.e., 1) (Hadfield 2010b).

### (Co)variance structure between endocrine traits and with behavior

To validate the assumption that waterborne steroid levels were representative of biological processes, we first estimated the correlations between the water borne and entire body levels of cortisol (F) and 11KT from the sacrificed fish (*n* = 26). Correlations were estimated between natural-log-transformed rates of hormone release scaled by mass. Following this, to characterize patterns of variance and covariance in endocrine traits, mixed model analyses similar to those described above were applied to the (natural-log-transformed) endocrine traits collected across the five trials, expressed in standard deviation units. For these analyses, rather than dividing by mass, we included mass as an additional fixed effect for all endocrine traits. Thus, we tested for repeatable variation in pre- (_PRE_) and the poststressor (_POST_) hormone levels of F and 11KT, estimated the covariance structure among these endocrine traits and partitioned it into within- and among-individual components as for the behavioral traits above.

To test the primary hypothesis predicted by the SCS paradigm, that among-individual differences in behavior are correlated with among-individual differences in endocrine physiology, we finally fitted additional multivariate models to estimate the among-individual correlation (*r*_I_) between endocrine and behavioral traits (ACT, emREF). Note that activity (ACT, percentage time active) was used here as a univariate proxy for baseline behavioral variation based on the eigen decomposition of the **I** matrix between behaviors (see Results below for details).

## Results

### Among-individual variance in behavior

Across the full set of baseline behavior traits, there was evidence for significant among-individual variance (comparison of models 1 & 2, 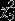 = 32.9, *P* < 0.001), as well as covariance structure among traits (model 2 vs. 3, 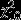 = 851.4, *P* < 0.001) that included an among-individual component (model 3 vs. 4, 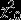 = 22.6, *P* = 0.013). Thus, we conclude that these behavioral traits are repeatable and covary among-individuals. From model 4, repeatabilities (SE) for baseline behaviors ranged from 0.101 (±0.105) for time in middle to 0.305 (±0.153) for activity (Table1a). Univariate analyses, assuming the test statistic to be asymptotically distributed as a mix of 50:50 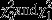 (following Visscher 2006), were statistically significant at *P* < 0.05 for all individual traits except time in middle (see Supporting Information materials, Table S1). Fixed effects estimated from these univariate models are also presented in Supporting Information materials for completeness (Table S2). Although the fixed effect results are of little direct relevance to the present objectives, we note there was little evidence of significant change in mean behavioral traits with trial number (Table S2 and Fig. S1), providing limited evidence of habituation and/or learning (but see Discussion for more details on this subject).

**Table 1:** Estimated R (residual, within-individual) and I (among-individual) matrices for (a) all baseline behavioral traits, (b) all endocrine traits and (c) Pretrial endocrine traits and activity (used a univariate proxy for boldness; see text). Trait-specific variances are shown on the diagonal (shaded), with between-trait covariances (below diagonal) and correlations (above diagonal). Variances on the diagonal of I can be interpreted as repeatabilities as (transformed) traits were scaled to standard deviation units. Standard errors are provided in parentheses

(a)					
	TL	ACT	AC	TIM	TOR
**R**					
Track length (TL)	0.722 (0.118)	0.984 (0.004)	0.913 (0.02)	0.632 (0.070)	0.942 (0.014)
Activity (ACT)	0.696 (0.115)	0.695 (0.114)	0.901 (0.022)	0.663 (0.065)	0.961 (0.009)
Area covered (AC)	0.680 (0.116)	0.658 (0.113)	0.769 (0.125)	0.801 (0.042)	0.881 (0.026)
Time in middle (TIM)	0.502 (0.107)	0.516 (0.107)	0.656 (0.120)	0.872 (0.141)	0.672 (0.064)
Time out of refuge (TOR)	0.681 (0.114)	0.682 (0.113)	0.658 (0.114)	0.534 (0.109)	0.726 (0.118)
**I**					
Track length (TL)	0.274 (0.145)	0.986 (0.011)	0.975 (0.034)	0.838 (0.249)	0.959 (0.034)
Activity (ACT)	0.285 (0.148)	0.305 (0.153)	0.957 (0.046)	0.902 (0.223)	0.992 (0.013)
Area covered (AC)	0.237 (0.134)	0.246 (0.136)	0.217 (0.131)	0.855 (0.184)	0.931 (0.064)
Time in middle (TIM)	0.140 (0.106)	0.158 (0.111)	0.127 (0.106)	0.101 (0.105)	0.927 (0.205)
Time out of refuge (TOR)	0.253 (0.139)	0.277 (0.145)	0.219 (0.130)	0.149 (0.108)	0.256 (0.141)

(b)				
	*F*_PRE_	11KT_PRE_	*F*_POST_	11KT_POST_
**R**				
Pretrial cortisol (*F*_PRE_)	0.594 (0.097)	0.051 (0.116)	0.066 (0.115)	−0.205 (0.111)
Pretrial 11-ketotestosterone (11KT_PRE_)	0.030 (0.069)	0.589 (0.097)	0.104 (0.115)	0.083 (0.115)
Post-trial cortisol (*F*_POST_)	0.049 (0.085)	0.076 (0.085)	0.903 (0.147)	0.356 (0.101)
Post-trial 11-ketotestosterone (11KT_POST_)	−0.138 (0.080)	0.056 (0.078)	0.296 (0.102)	0.766 (0.124)
**I**				
Pretrial cortisol (*F*_PRE_)	0.091 (0.077)	0.768 (0.389)	0.854 (1.102)	0.881 (1.284)
Pretrial 11-ketotestosterone (11KT_PRE_)	0.104 (0.071)	0.202 (0.113)	0.552 (0.807)	0.867 (0.872)
Post-trial cortisol (*F*_POST_)	0.051 (0.059)	0.049 (0.072)	0.039 (0.087)	0.815 (1.210)
Post-trial 11-ketotestosterone (11KT_POST_)	0.054 (0.056)	0.078 (0.071)	0.033 (0.064)	0.041 (0.081)

(c)			
	*F*_PRE_	11KT_PRE_	ACT
**R**			
Pretrial cortisol (*F*_PRE_)	0.594 (0.097)	0.056 (0.116)	−0.026 (0.116)
Pretrial 11-ketotestosterone (11KT_PRE_)	0.033 (0.069)	0.591 (0.098)	−0.052 (0.116)
Activity (ACT)	−0.017 (0.075)	−0.034 (0.075)	0.697 (0.115)
**I**			
Pretrial cortisol (*F*_PRE_)	0.090 (0.076)	0.743 (0.396)	0.785 (0.391)
Pretrial 11-ketotestosterone (11KT_PRE_)	0.099 (0.070)	0.198 (0.111)	0.383 (0.350)
Activity (ACT)	0.129 (0.081)	0.093 (0.094)	0.300 (0.151)

**Figure 2:**
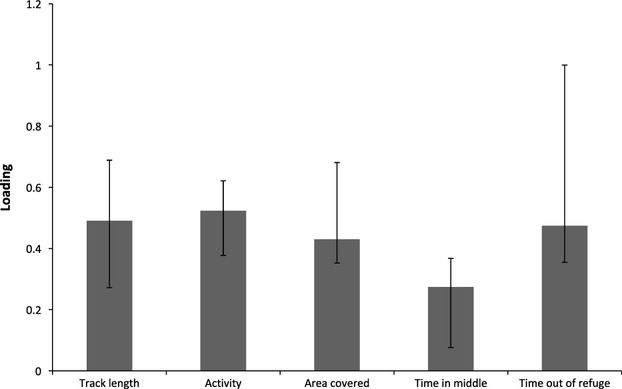
Loadings (in Standard Deviation units) on the first eigen vector of I, representing 96.2% of the total estimated variance for the baseline behavior traits. Error bars indicate 95% highest probability density intervals estimated by parametric bootstrap (see text for details).

Between baseline traits, the among-individual correlations (*r*_I_) were positive and strong, ranging from 0.838 (±0.249) between track length and time in middle, to 0.986 (±0.011) between track length and activity (Table1a). Consistent with this correlation structure, we found that 96.2% of the variance in **I** was explained by the first eigenvector of **I** (Fig.2, Supporting Information Table S3). Trait loadings on this vector are all significantly positive (as bootstrapped 95% confidence intervals do not span zero) and are broadly similar in magnitude (Fig.2). This means that, commensurate with our *a priori* expectations of boldness, individuals with consistently higher track length are also (consistently) more active, cover greater area, and spend more time in the middle of the arena and more time out of the refuge. This result provides independent experimental confirmation of our previous finding that a strong axis of boldness variation exists in this population (Boulton et al. 2014).

Statistical support for among-individual variance in tendency to emerge after the acute stressor (predator strike) was less compelling. Using MCMCglmm, the estimated repeatability for emREF (on the liability scale) was moderately high (intraclass correlation (IC) = 0.406, 95% higher probability density (HPD) 0.074–0.790). Note, however, that this estimate (and so the related HPD interval) is constrained to be positive in the analysis such that this does not necessarily equate to a “significant” result in frequentist terms, and arguably, the posterior mode of IC was not clearly distinct from zero (Fig. S2). For comparison, we estimated a repeatability (SE) for emREF on the observed scale of 0.160 (±0.107) using REML. Although nominally significant (*P* = 0.04; see Table S1), the likelihood ratio test applied makes an assumption of residual normality that is clearly violated as this is a binary trait. MCMCglmm estimates of *r*_I_ (95% CI) between emREF and baseline behaviors were all positive but not statistically significant, ranging from 0.172 (−0.479–0.830) for track length to 0.508 (−0.452–0.839) for activity (Table2). Taking these results together, we interpret variation in emREF cautiously. Some variance among individuals in response to the acute stressor appears to be present but does not have unequivocal statistical support. Accepting the premise that individuals do differ, those individuals that are more likely to re-emerge following the simulated predator strike tend to be the bolder fish, as indicated by baseline behaviors. However, this qualitative pattern is not statistically robust in our data.

**Table 2:** MCMCglmm estimates of intraclass correlations (*r*_I_) between prestrike behaviors and poststrike Emergence, with 95% upper and lower higher probability density values

		95% HPD interval	
Emergence with	*r*_I_	Lower	Upper
Track length	0.172	−0.479	0.830
Activity	0.508	−0.452	0.839
Area covered	0.337	−0.421	0.930
Time in middle	0.279	−0.639	0.962
Time out of refuge	0.214	−0.599	0.827

### Among-individual variance in endocrine traits

**Figure 3:**
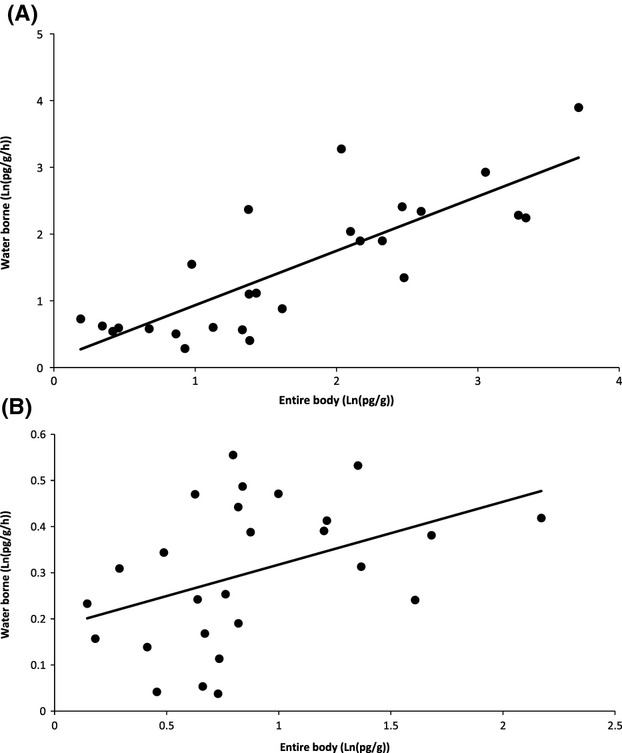
Relationships between water borne and entire body levels of (A) cortisol and (B) 11-ketotestosterone (11KT). Solid lines show ordinary least squares regressions.

Our validation sample confirmed significant positive correlations (r) between mass-adjusted waterborne release rate and whole-body hormone concentrations. For cortisol, the relationship was strong (*r* = 0.815, ±0.067, *P* < 0.001) and linear on a (natural) log–log scale (Fig.3A). For 11KT, the relationship was weaker, but nonetheless positive and significantly greater than zero (*r* = 0.420 ± 0.165, *P* = 0.028; Fig.3B). Thus, we consider waterborne endocrine levels to be an appropriate proxy for whole-body measures in this species. In our experimental samples, absolute cortisol release rates were actually higher in the pre- than poststressor collection periods (mean *F*_PRE_ (SE) = 1871 (±176) pg/h, mean *F*_POST_ (SE) = 669 (±64.9) pg/h). Comparison of paired samples confirmed that individuals released significantly less cortisol in the post-trial collection period (paired sample *t*-test, *t*_98_ = 7.17, *P* < 0.001). There was no evidence for a difference in 11KT levels between pre- and postsampling periods (premean (SE) = 105.56 (±4.21) pg/h, postmean (SE) = 99.69 (±3.63) pg/h, paired sample *t*-test, *t*_96_ = 1.169, *P* = 0.123).

Multivariate models provided evidence of among-individual variance in endocrine phenotype (comparison of models 1 & 2, 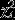 = 9.57, *P* = 0.048). Covariance between traits was also present (model 2 vs. 3, 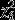 = 21.6, *P* = 0.001), although an among-individual component to this was not statistically supported (model 3 vs. 4, 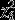 = 5.83, *P* = 0.443). Under the full model (4), repeatabilities (SE) varied from 0.039 (±0.087) for *F*_POST_ to 0.202 (±0.113) for 11KT_PRE_ (Table1b). Univariate models yielded similar repeatability estimates (Table S1) and revealed significant effects of *day order* (for 11KT, but not F) and *trial number* (for all endocrine measures except *F*_POST_) (Table S2). Although this suggests the potential for an effect of habituation and/or learning on endocrine state, there was no evidence of simple linear pattern across *trial number* (Fig. S1B). Regardless of cause, we note that including this fixed effect yields repeatability estimates that are controlled (statistically) for *trial number* effects on the trait mean. *V*_**I**_ was only statistically significant for 11KT_PRE_. Thus, we conclude that robustly supported among-individual variance in endocrine state is limited to 11KT_PRE_, although we note that the estimate of *V*_I_ for *F*_PRE_ was marginally nonsignificant in the univariate analysis.

Examination of among- and within–individual matrices (**I** and **R**) between endocrine traits (Table1b) showed that the significant covariance structure detected was likely driven by a single positive relationship between *F*_POST_ and 11KT_POST_. 90% of the covariance between these traits was partitioned into **R**, yielding a within-individual correlation (*r*_R_ [SE]) of 0.356 (±0.101). Given no evidence of among-individual significant covariance structure in **I,** we do not further consider pairwise estimates of correlations (*r*_I_) except to note that the estimate between *F*_PRE_ and 11KT_PRE_ was strongly positive and approaching significance (*r*_I_ = 0.768 [±0.389]). Thus to the extent that F_PRE_ is actually repeatable (see later Discussion), individuals with higher cortisol release rates are also characterized by higher androgen levels, not lower as we expected *a priori*.

### Correlation structure between activity, F and 11KT

Finally, to test among-individual correlation (*r*_I_) between boldness and endocrine state, we fitted trivariate models of activity (ACT), *F*_PRE_ and 11KT_PRE_. We chose to use ACT as a univariate proxy for boldness given the strong correlation structure in **I** among baseline behaviors and since ACT has the highest loading (with the narrowest confidence interval) on the estimated vector of boldness (see above, Table1a and Fig.2). This simplifies the analysis and allows us to avoid the issue of carrying forward uncertainty associated with multivariate predictors of boldness (e.g., generated from principal component scores or similar estimates). *F*_POST_ and 11KT_POST_ were not included in these multivariate models given the lack of repeatable variation for these traits. Model comparisons confirmed among-individual variance (model 1 vs. 2, 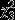 = 17.3, *P* < 0.001); however, the model was not significantly improved by inclusion of within- (**R**) or among-individual (**I**) between-trait covariance (model 2 vs. 3, 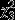 = 0.086, *P* = 0.848; model 3 vs. 4, 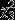 = 6.98, *P* = 0.073). Under Model 4, estimated repeatabilities were similar to those already reported (Table1c). While reiterating that our model comparisons indicate nonsignificant between-trait covariance structure (within- and among-individuals), our r_I_ estimates are positive and strong in some cases (Table1c). Thus, we find no support for a negative *r*_I_ between boldness and cortisol levels as predicted under the SCS model. Rather, the qualitative result is that, counter to our expectations, individuals characterized by higher (prestressor) release rates of F and 11KT are the bolder individuals as measured by ACT.

## Discussion

Overall, our results provide limited support for among-individual (co)variation consistent with an integrated stress-coping style (SCS) in *Xiphophorus birchmanni*. Individuals did differ consistently in their behavioral responses to mild stress imposed by the modified open field trial. Furthermore, this behavioral variation is consistent with an underlying shy–bold axis of personality. However, it is less clear that individuals differ significantly in behavioral response to the simulated predator attack. Additionally, while there is some evidence of repeatable variation in endocrine state, robust statistical support was limited to pretrial 11KT levels. Although not statistically significant, there was a tendency for bolder or more behaviorally proactive individuals to release more cortisol. Although potentially indicative of some degree of integration between behavioral and endocrine stress response components, this pattern is actually counter to the SCS model's prediction of lower HPA/HPI activity in proactive individuals (Koolhaas et al. 1999). In what follows, we discuss first the behavioral, and then the endocrine data in more detail before commenting further on the relationship between the two. In addition to presenting our biological conclusions, we also highlight a number of methodological issues and difficulties of interpretation that warrant further consideration.

We found partial support for our first hypothesis that fish would differ consistently in behavioral response to stress exposure. Analysis of behavioral data collected under the mild stress stimulus showed that individual traits assayed were repeatable, and the **I** matrix contained significant among-individual correlation structure consistent with a single latent axis (or personality trait) underpinning the observed variation. Moving along this axis, hereafter interpreted as shyness–boldness, trait expression changes in a concerted manner. Thus a fish that consistently swims further is also more active explores a greater area, spends more time in the center of the experimental arena, and spends less time hiding in the refuge. This finding confirms our earlier report of a strong axis of boldness variation in *Xiphophorus birchmanni* that is broadly stable over long time periods (i.e., representative of lifespan under natural conditions Boulton et al. 2014) and adds to rapidly accumulating evidence of personality variation in fishes (Burns 2008; Toms et al. 2010; Wilson et al. 2013). However, we note that our data do not clearly support the expectation that boldness (as inferred from the baseline data) leads to faster re-emergence following the moderately stressful simulated predation event. To some extent, this could reflect a lack of statistical power caused by reliance on the binary emREF variable and we acknowledge that a longer poststrike observation period (to avoid censoring latency to emerge) may have afforded greater biological insights by giving access to more detailed information on subsequent behavioral variation. Nonetheless, our findings do highlight an interesting question for future empirical studies: To what extent are among-individual behavioral stress response profiles consistent across stress stimuli of varying type or intensity?

Our second hypothesis regarding repeatable among-individual variation of endocrine state also was supported only partially. We found significant variation among individuals for pretrial androgen levels, with a repeatability of approximately 10%. However, the repeatability of pretrial cortisol levels was only half that and (marginally) nonsignificant. We found no support whatsoever for repeatable variation of either *F*_POST_ or 11KT_POST_. Note that we analyzed pre- and post-trial hormone levels rather than defining the change (i.e., response) as the trait of interest, as reducing two traits to one inevitably leads to a loss of information. Nonetheless, consideration of the response offers a complementary and intuitive viewpoint. Additional models (results not shown) provided no statistical evidence of repeatable variation in endocrine responses, defined as the log-transformed postminus log-transformed prehormone release rates.

Repeatabilities of labile traits are typically expected to decline with the interobservation time period (Bell et al. 2009) and/or the total period of time that observations are made over (Boulton et al. 2014). Given that the repeatability of *F*_PRE_ was approaching significance, we carried out additional *post hoc* analysis that revealed significant (positive) correlations among trial specific measures (Table3), being strongest between successive trials in the first half of the study period (i.e., 1 and 2, 2 and 3). Consistent with this finding, fitting a univariate mixed model to data from the first three trials yielded a much higher repeatability for *F*_PRE_ than our estimates using all data (repeatability = 0.323 (±0.155), *P* = 0.027).

**Table 3:** Estimated between-trial (T1–T5) correlations of precontest cortisol levels. Estimates are conditioned on effects of weight and day order. Standard errors are shown in parentheses and significant correlations (inferred from |*r*|≥2SE) are denoted by bold font

	T1	T2	T3	T4
T2	**0.845** (0.074)			
T3	**0.521** (0.191)	**0.717** (0.142)		
T4	**0.562** (0.180)	**0.530** (0.197)	0.323 (0.229)	
T5	−0.213 (0.269)	−0.314 (0.274)	−0.297 (0.275)	−0.022 (0.262)

Thus, we conclude that there are some real differences among individuals in pretrial cortisol synthesis but that, relative to 11KT_PRE_ (and baseline behaviors as discussed above), these differences were less stable over the time course of our study. Our study does not address the biological reasons why this may be the case, although Table3 indicates that the relatively low estimate of repeatability overall is driven particularly by a lack of correlation between trial 5 and other observations. We note that significant effects of *Trial* on mean *F*_PRE_ were detected (Table S2), with an initial increase from trials 1 to 3 (Fig. S1B) followed by a decline across the final two observations. This is potentially indicative of habituation (on average) to stress caused by the endocrine assay procedure itself, or to an increase in the rate of negative feedback resulting in a decrease rate of cortisol output (Wong et al. 2008; Fischer et al. 2014; see Discussion below). Although the inclusion of *trial number* as a fixed effect in the models controls for the average effect of any habituation process, if the degree or rate of habituation or change in rate of negative feedback differs among individuals then this could contribute to the low correlations between F_PRE_ at trial 5 and the earlier observations.

Our third hypothesis was that behavioral and physiological stress response pathways would be integrated within individuals. Specifically, under the SCS model, we predicted bolder individuals would be characterized by consistently lower glucocorticoid release but higher androgen levels (Earley and Hsu 2008; Glenn et al. 2011). Statistical support for among-individual covariance in our trivariate analysis of boldness (activity), *F*_PRE_ and 11KT_PRE_ was marginally nonsignificant but, in light of our conclusion that some among-individual variation in *F*_PRE_ is present, we consider two aspects of the estimated correlation structure to be noteworthy. Firstly, the among-individual correlation (*r*_I_) between *F*_PRE_ and 11KT_PRE_ was strongly positive. Although within- and between-individual covariance cannot be partitioned from a single observation, it was also the case that (mass adjusted) levels of the two hormones were positively correlated in validation samples (water borne *r* = 0.624 (0.122), *P* < 0.001; entire body *r* = 0.846 (0.047), *P* < 0.001). Thus, while we had predicted a negative relationship between (repeatable) levels of cortisol and 11KT, our results actually point toward it being positive. Secondly, we found a strong positive among-individual correlation (*r*_I_) between activity and *F*_PRE_. Thus, it is the bold (or proactive) behavioral types that exhibit higher rates of glucocorticoid release prior to undergoing the trial, counter to the predictions of the SCS model. This finding is concurrent with a recent study in *Xiphophorus helleri* (Boulton et al. 2012), although in that case, a lack of repeated measures meant we were unable to exclude the possibility of the relationship being driven by trial-(as opposed to individual-) specific effects.

A number of empirical studies have reported negative correlations between bold or proactive behaviors and HPA/HPI activity consistent with predictions of the SCS model, although most of these studies have used only a single observation per subject (Sloman et al. 2001; Brown et al. 2005; Verbeek et al. 2008; Raoult et al. 2012). However, exceptions to this pattern are also found, particularly in studies that have used repeated measures to quantify relationships at the among-individual level (e.g., Van Reenen et al. 2005; Ferrari et al. 2013). The present results therefore add further weight to the suggestion that the SCS model, at least as originally proposed, may be overly simplistic (Koolhaas et al. 2010). One possibility is that a model with two (or more) independent axes of behavioral response variation, for example, locomotion and fearfulness (Van Reenen et al. 2005; Ferrari et al. 2013), might be more appropriate. Equally, this may be true for endocrine response, with variation in the degree of the endocrine response, habituation and negative feedback all having the potential to be independent axes of endocrine response variation. Recently, an argument has been put forward that distinguishing between the qualitative (coping style) and quantitative (stress reactivity) components of among-individual variation is important (Koolhaas et al. 2010). Koolhaas et al. (2010) also suggest that widespread support for the proactive–reactive SCS model in domesticated species may be an artifact of strong selection on either physiological or behavioral traits in captive-bred populations. If so then, relationships between these traits will likely be more variable in wild populations. Although the fish used in our study were captive bred, they were only two generations removed from the wild and can therefore be considered broadly genetically representative of their natural source population.

The waterborne endocrine assay has been verified in many fishes including a number of Poeciliids, (e.g., Netherton et al. 2004; Archard et al. 2012; Gabor and Contreras 2012). Here, we were able to validate its use as a noninvasive proxy for whole-body hormone levels in the sheepshead swordtail, *Xiphophorus birchmanni*. Nonetheless, some patterns in our data pose challenges for interpretation. In particular, we found a significant decline in mean cortisol released between paired (i.e., individual and trial specific) pre- and post-trial samples. Thus on average, the cortisol “response” to stress imposed by the trial was negative, not positive as expected. It is possible that our 60-min steroid collection period was too long resulting in capture of the cortisol surge released as a result of handling stress in the F_PRE_ levels, and saturation of the HPI axis due to negative feedback and/or reabsorption of cortisol during the F_POST_ collection (Scott and Ellis 2007). Arguments that waterborne collection procedures are stressful, despite being noninvasive, have been put forward (Wong et al. 2008). Thus, rather than being “baseline” measures, our F_PRE_ may indeed be indicative of a stress response. There have also been suggestions of habituation to the technique, rendering the repeated measures approach difficult to interpret (Wong et al. 2008; Fischer et al. 2014). Here, we found significant changes in mean F_PRE_ levels across trials (with an initial increase followed by declining levels after the third trial, Fig. S1B). Suggestions that a “flow-through” system for steroid collection may be a better method of hormone collection as fish do not then encounter confinement stress are valid (Scott and Ellis 2007); however, necessarily waterborne collection requires physical and chemical isolation, and, if studies on both behavioral and physiological components of SCS are to be carried out, then these necessitate capture, handling, and confinement.

In summary, our multivariate repeated measures approach allowed us to characterize physiological and behavioral response to an acute stressor in a second-generation captive-bred population of *X. Birchmanni*. Although there was evidence for among-individual variance in behaviors and 11KT, the lack of significant repeatability (over the full experiment) for cortisol and the positive correlations between physiological and behavioral traits did not lend support to the SCS paradigm. The fact that repeatabilities of endocrine levels were stronger when observations were closer together suggests the potential for experimental design to have a strong influence on biological conclusions regarding whether or not a trait is repeatable. Our findings add weight to the suggestion that cortisol measures in wild (or recently wild derived) populations may be less stable than those measured in laboratory adapted populations (Koolhaas et al. 2010). In line with other recent studies, our results also suggest that the waterborne collection procedure used is a mild stressor, and thus that interpretation of these pretrial levels as “baseline” levels may not be appropriate. We therefore conclude that the stress-coping style model is not well supported in this species, as physiological and behavioral responses do not clearly covary along a single axis of latent variation among individuals. Determining whether or not this finding is generally true across species and/or environmental contexts will require further studies and, crucially, wider adoption of repeated measures designs to allow within- and between-individual sources of covariation to be disentangled.

## Ethical Statement

The University of Exeter local ethical review committee approved all work in this study that was carried out under license granted by the Home Office (UK) under the Animals (Scientific Procedures) Act 1986.

## Supporting Information

**Table S1.**Estimates of among-individual (*V*_I_) and residual (*V*_R_) variance for all traits with standard errors in parentheses. **Table S2.** Estimated fixed effects from univariate mixed models of all traits. **Table S3.** Eigen vector decomposition of the **I** matrix estimated among behavioural traits observed in the modified open field trial prior to the simulated predator attack. **Figure S1.** Estimated effects of trial number (*Trial*) from univariate models of (A) behavioural and (B) endocrine traits (see Supporting Information Tables S1 and S2 for full results). **Figure S2.** Posterior distribution of the intraclass correlation (IC) of the binary trait, emergence from refuge (emREF) from an analysis modelling emREF as a categorical trait in MCMCglmm.

## References

[b1] Andrade O, Orihuela A, Solano J, Galina CS (2001). Some effects of repeated handling and the use of a mask on stress responses in Zebu cattle during restraint. Appl. Anim. Behav. Sci.

[b2] Archard GA, Braithwaite VA (2011). Increased exposure to predators increases both exploration and activity level in *Brachyrhaphis episcopi*. J. Fish Biol.

[b3] Archard GA, Earley RL, Hanninen AF, Braithwaite VA (2012). Correlated behaviour and stress physiology in fish exposed to different levels of predation pressure. Funct. Ecol.

[b4] Barber I, Walker P, Svensson PA (2004). Behavioural responses to simulated avian predation in female three spined sticklebacks: the effect of experimental *Schistocephalus solidus* infections. Behaviour.

[b5] Barreto RE, Volpato GL (2011). Ventilation rates indicate stress-coping styles in Nile tilapia. J. Biosci.

[b6] Barton BA (2002). Stress in fishes: a diversity of responses with particular reference to changes in circulating corticosteroids. Integr. Comp. Biol.

[b7] Bell AM, Hankison SJ, Laskowski KL (2009). The repeatability of behaviour: a meta-analysis. Anim. Behav.

[b8] Benus RF, Bohus B, Koolhaas JM, Vanoortmerssen GA (1991). Heritable variation for aggression as a reflection of individual coping strategies. Experientia.

[b9] Blanchard RJ, Nikulina JN, Sakai RR, Mckittrick C, Mcewen B, Blanchard DC (1998). Behavioral and endocrine change following chronic predatory stress. Physiol. Behav.

[b10] Blas J, Bortolotti GR, Tella JL, Baos R, Marchant TA (2007). Stress response during development predicts fitness in a wild, long lived vertebrate. Proc. Natl Acad. Sci. USA.

[b11] Borg B, Mayer I (1995). Androgens and behaviour in the three-spined stickleback. Behaviour.

[b12] Boulton K, Massault C, Koning DJD, Houston R, Haley C, Batargias C (2011). QTL for growth and stress response in the gilthead seabream (*Sparus aruata*. Aquaculture.

[b13] Boulton K, Sinderman B, Pearce M, Earley R, Wilson A (2012). He who dares only wins sometimes: physiological stress and contest behaviour in *Xiphophorus helleri*. Behaviour.

[b14] Boulton K, Grimmer AJ, Rosenthal GG, Walling CA, Wilson AJ (2014). How stable are personalities? A multivariate view of behavioural variation over long and short timescales in the sheepshead swordtail, *Xiphophorus birchmanni*. Behav. Ecol. Sociobiol.

[b15] Breuner CW, Patterson SH, Hahn TP (2008). In search of relationships between the acute adrenocortical response and fitness. Gen. Comp. Endocrinol.

[b16] Briffa M, Rundle SD, Fryer A (2008). Comparing the strength of behavioural plasticity and consistency across situations: animal personalities in the hermit crab *Pagurus bernhardus*. Proc. R. Soc. B Biol. Sci.

[b17] Brown C, Gardner C, Braithwaite VA (2005). Differential stress responses in fish from areas of high- and low-predation pressure. J. Comp. Physiol. B.

[b18] Brown C, Burgess F, Braithwaite VA (2007). Heritable and experiential effects on boldness in a tropical poeciliid. Behav. Ecol. Sociobiol.

[b19] Budaev SV (1997). “Personality” in the guppy (*Poecilia reticulata*): a correlational study of exploratory behavior and social tendency. J. Comp. Psychol.

[b20] Budaev SV, Zworykin DD, Mochek AD (1999). Consistency of individual differences in behaviour of the lion-headed cichlid, *Steatocranus casuarius*. Behav. Process.

[b21] Burns JG (2008). The validity of three tests of temperament in guppies (*Poecilia reticulata*. J. Comp. Psychol.

[b22] Carere C, Groothuis TGG, Mostl E, Daan S, Koolhaas JM (2003). Fecal corticosteroids in a territorial bird selected for different personalities: daily rhythm and the response to social stress. Horm. Behav.

[b23] Carere C, Caramaschi D, Fawcett TW (2010). Covariation between personalities and individual differences in coping with stress: converging evidence and hypotheses. Curr. Zool.

[b24] Castanheira MF, Herrera M, Costas B, Conceição LEC, Martins CIM (2013). Can we predict personality in fish? Searching for consistency over time and across contexts. PLoS ONE.

[b25] Chrousos GP (1998). Stressors, stress, and neuroendocrine integration of the adaptive response – The 1997 Hans Selye Memorial Lecture. Stress of Life.

[b26] Connor-Smith JK, Flachsbart C (2007). Relationships between personality and coping: a meta-analysis. J. Pers. Soc. Psychol.

[b27] Creel S (2001). Social dominance and stress hormones. Trends Ecol. Evol.

[b28] Devries AC (2002). Interaction among social environment, the hypothalamic-pituitary-adrenal axis, and behavior. Horm. Behav.

[b29] Dingemanse NJ, Dochtermann NA (2013). Quantifying individual variation in behaviour: mixed-effect modelling approaches. J. Anim. Ecol.

[b30] Dingemanse NJ, Dochtermann NA, Wright J (2010). A method for exploring the structure of behavioural syndromes to allow formal comparison within and between data sets. Anim. Behav.

[b31] Dochtermann NA, Roff DA (2010). Applying a quantitative genetics framework to behavioural syndrome research. Proc. R. Soc. B Biol. Sci.

[b32] Earley RL (2006). *Xiphophorus*: carving a niche towards a broader understanding of aggression and dominance. Zebrafish.

[b33] Earley RL, Hsu Y (2008). Reciprocity between endocrine state and contest behavior in the killifish, *Kryptolebias marmoratus*. Horm. Behav.

[b34] Ellis T, James JD, Stewart C, Scott AP (2004). A non-invasive stress assay based upon measurement of free cortisol released into the water by rainbow trout. J. Fish Biol.

[b35] Ellis T, James JD, Sundh H, Fridell F, Sundell K, Scott AP (2007). Non-invasive measurement of cortisol and melatonin in tanks stocked with seawater Atlantic salmon. Aquaculture.

[b36] Evans JP, Gasparini C, Pilastro A (2007). Female guppies shorten brood retention in response to predator cues. Behav. Ecol. Sociobiol.

[b37] Ferrari C, Pasquaretta C, Carere C, Cavallone E, Von Hardenberg A, Reale D (2013). Testing for the presence of coping styles in a wild mammal. Anim. Behav.

[b38] Fevolden SE, Refstie T, Gjerde B (1993). Genetic and phenotypic parameters for cortisol and glucose stress response in Atlantic salmon and Rainbow trout. Aquaculture.

[b39] Fischer EK, Harris RM, Hofmann HA, Hoke KL (2014). Predator exposure alters stress physiology in guppies across timescales. Horm. Behav.

[b40] Fraisse F, Cockrem JF (2006). Corticosterone and fear behaviour in white and brown caged laying hens. Br. Poult. Sci.

[b41] Gabor CR, Contreras A (2012). Measuring water-borne cortisol in *Poecilia latipinna*: is the process stressful, can stress be minimized and is cortisol correlated with sex steroid release rates?. J. Fish Biol.

[b42] Gilmour AR, Gogel BJ, Cullis BR, Thompson R (2009). ASReml user guide release 3.0.

[b43] Glenn AL, Raine A, Schug RA, Gao Y, Granger DA (2011). Increased testosterone-to-cortisol ratio in psychopathy. J. Abnorm. Psychol.

[b44] Gosling SD, John OP (1999). Personality dimensions in nonhuman animals: a cross-species review. Curr. Dir. Psychol. Sci.

[b45] Hadfield JD (2010a). MCMC methods for multi-response generalized linear mixed models: the MCMCglmm R package. J. Stat. Softw.

[b46] Hadfield JD (2010b). http://cran.r-project.org/web/packages/MCMCglmm/vignettes/CourseNotes.pdf.

[b47] Hessing MJC, Hagelso AM, Schouten WGP, Wiepkema PR, Vanbeek JAM (1994). Individual behavioural and physiological strategies in pigs. Physiol. Behav.

[b48] Huntingford FA (1976). Relationship between anti-predator behavior and aggression among conspecifics in 3-spined stickleback, *Gasterosteus aculeatus*. Anim. Behav.

[b49] Huntingford FA, Andrew G, Mackenzie S, Morera D, Coyle SM, Pilarczyk M (2010). Coping strategies in a strongly schooling fish, the common carp *Cyprinus carpio*. J. Fish Biol.

[b50] Johnson EO, Kamilaris TC, Chrousos GP, Gold PW (1992). Mechanisms of stress – a dynamic overview of hormonal and behavioral homeostasis. Neurosci. Biobehav. Rev.

[b51] Kime DE, Manning NJ (1982). Seasonal patterns of free and conjugated androgens in the brown trout *Salmo trutta*. Gen. Comp. Endocrinol.

[b52] Koolhaas JM, Deboer SF, Bohus B (1997). Motivational systems or motivational states: behavioural and physiological evidence. Appl. Anim. Behav. Sci.

[b53] Koolhaas JM, Korte SM, De Boer SF, Van Der Vegt BJ, Van Reenen CG, Hopster H (1999). Coping styles in animals: current status in behavior and stress-physiology. Neurosci. Biobehav. Rev.

[b54] Koolhaas JM, De Boer SF, Coppens CM, Buwalda B (2010). Neuroendocrinology of coping styles: towards understanding the biology of individual variation. Front. Neuroendocrinol.

[b55] Koolhaas JM, Bartolomucci A, Buwalda B, De Boer SF, Flugge G, Korte SM (2011). Stress revisited: a critical evaluation of the stress concept. Neurosci. Biobehav. Rev.

[b56] Korte SM, Koolhaas JM, Wingfield JC, Mcewen BS (2005). The Darwinian concept of stress: benefits of allostasis and costs of allostatic load and the trade-offs in health and disease. Neurosci. Biobehav. Rev.

[b57] Levine S, Brown MR, Koob GF, River C, Ursine H (1991). What is stress?. Stress: neurobiology and neuroendocrinology.

[b58] Massault C, Hellemans B, Louro B, Batargias C, Van Houdt JKJ, Canario A (2010). QTL for body weight, morphometric traits and stress response in European sea bass *Dicentrarchus labrax*. Anim. Genet.

[b59] Mayer I, Borg B, Schulz R (1990). Seasonal changes in and effect of castration/androgen replacement on the plasma-levels of five androgens in the male three-spined stickleback, *Gasterosteus aculeatus* L. Gen. Comp. Endocrinol.

[b60] Mommsen TP, Vijayan MM, Moon TW (1999). Cortisol in teleosts: dynamics, mechanisms of action, and metabolic regulation. Rev. Fish Biol. Fisheries.

[b61] Netherton JD, Grober MS, Earley RL (2004). Temporal decay of cortisol in green swordtail fish (*Xiphophorus helleri*) following aggressive encounters: differences between winners and losers?. Horm. Behav.

[b62] Oliveira RF, Hirschenhauser K, Carneiro LA, Canario AVM (2002). Social modulation of androgen levels in male teleost fish. Comp. Biochem. Physiol. B Biochem. Mol. Biol.

[b63] Øverli Ø, Sorensen C, Pulman KGT, Pottinger TG, Korzan WJ, Summers CH (2007). Evolutionary background for stress-coping styles: relationships between physiological, behavioral, and cognitive traits in non-mammalian vertebrates. Neurosci. Biobehav. Rev.

[b64] Pellis SM, Mckenna MM (1992). Intrinsic and extrinsic influences on play fighting in rats – effects of dominance, partners playfulness, temperament and neonatal exposure to testosterone propionate. Behav. Brain Res.

[b65] Pickering AD, Pottinger TG (1989). Stress responses and disease resistance in salmonid fish – effects of chronic elevation of plasma-cortisol. Fish Physiol. Biochem.

[b66] Pottinger TG (2010). A multivariate comparison of the stress response in three salmonid and three cyprinid species: evidence for inter-family differences. J. Fish Biol.

[b67] Pottinger TG, Carrick TR (1999). Modification of the plasma cortisol response to stress in rainbow trout by selective breeding. Gen. Comp. Endocrinol.

[b68] Raoult V, Brown C, Zuberi A, Williamson JE (2012). Blood cortisol concentrations predict boldness in juvenile mulloway (*Argyosomus japonicus*. J. Ethol.

[b69] Réale D, Reader SM, Sol D, Mcdougall PT, Dingemanse NJ (2007). Integrating animal temperament within ecology and evolution. Biol. Rev.

[b70] Rudin FS, Briffa M (2012). Is boldness a resource-holding potential trait? Fighting prowess and changes in startle response in the sea anemone, *Actinia equina*. Proc. R. Soc. B Biol. Sci.

[b71] Scott AP, Ellis T (2007). Measurement of fish steroids in water – a review. Gen. Comp. Endocrinol.

[b72] Scott AP, Liley NR (1994). Dynamics of excretion of 17-alpha,20-beta-dihydroxy-4-pregnen-3-one 20-sulfate, and of the glucuronides of testosterone and 17-beta-estradiol, by urine of reproductively mature male and female rainbow-trout (*Oncorhynchus-mykiss*. J. Fish Biol.

[b73] Sebire M, Katsiadaki I, Scott AP (2007). Non-invasive measurement of 11-ketotestosterone, cortisol and androstenedione in male three-spined stickleback (*Gasterosteus aculeatus*. Gen. Comp. Endocrinol.

[b74] Selye H (1973). Evolution of stress concept. Am. Sci.

[b75] Sgoifo A, De Boer SF, Buwalda B, Korte-Bouws G, Tuma J, Bohus B (1998). Vulnerability to arrhythmias during social stress in rats with different sympathovagal balance. Am. J. Physiol. Heart Circ. Physiol.

[b76] Sih A, Bell A, Johnson JC (2004). Behavioral syndromes: an ecological and evolutionary overview. Trends Ecol. Evol.

[b77] Silberg J, Pickles A, Rutter M, Hewitt J, Simonoff E, Maes H (1999). The influence of genetic factors and life stress on depression among adolescent girls. Arch. Gen. Psychiatry.

[b78] Sloman KA, Metcalfe NB, Taylor AC, Gilmour KM (2001). Plasma cortisol concentrations before and after social stress in rainbow trout and brown trout. Physiol. Biochem. Zool.

[b79] Stram DO, Lee JW (1994). Variance-components testing in the longitudinal mixed effects model. Biometrics.

[b80] Sutherland MA, Huddart FJ (2012). The effect of training first-lactation heifers to the milking parlor on the behavioral reactivity to humans and the physiological and behavioral responses to milking and productivity. J. Dairy Sci.

[b81] Taves MD, Desjardins JK, Mishra S, Balshine S (2009). Androgens and dominance: sex-specific patterns in a highly social fish (*Neolamprologus pulcher*. Gen. Comp. Endocrinol.

[b82] Thaker M, Lima SL, Hews DK (2009). Acute corticosterone elevation enhances antipredator behaviors in male tree lizard morphs. Horm. Behav.

[b83] Thomson JS, Watts PC, Pottinger TG, Sneddon LU (2011). Physiological and genetic correlates of boldness: characterising the mechanisms of behavioural variation in rainbow trout, *Oncorhynchus mykiss*. Horm. Behav.

[b84] Toms CN, Echevarria DJ, Jouandot DJ (2010). A methodological review of personality-related studies in fish: focus on the shy-bold axis of behavior. Int. J. Comp. Psychol.

[b85] Van Reenen CG, O'connell NE, Van Der Werf JTN, Korte SM, Hopster H, Jones RB (2005). Responses of calves to acute stress: individual consistency and relations between behavioral and physiological measures. Physiol. Behav.

[b86] Veenema AH (2009). Early life stress, the development of aggression and neuroendocrine and neurobiological correlates: what can we learn from animal models?. Front. Neuroendocrinol.

[b87] Verbeek MEM, Boon A, Drent PJ (1996). Exploration, aggressive behavior and dominance in pair-wise confrontations of juvenile male great tits. Behaviour.

[b88] Verbeek P, Iwamoto T, Murakami N (2008). Variable stress-responsiveness in wild type and domesticated fighting fish. Physiol. Behav.

[b89] Visscher PM (2006). A note on the asymptotic distribution of likelihood ratio tests to test variance components. Twin Res. Hum. Genet.

[b90] Wechsler B (1995). Coping and coping strategies – a behavioural review. Appl. Anim. Behav. Sci.

[b91] Wendelaar Bonga SE (1997). The stress response in fish. Physiol. Rev.

[b92] Wesley RL, Cibils AF, Mulliniks JT, Pollak ER, Petersen MK, Fredrickson EL (2012). An assessment of behavioural syndromes in rangeland-raised beef cattle. Appl. Anim. Behav. Sci.

[b93] Wilson DS (1998). Adaptive individual differences within single populations. Philos. Trans. R. Soc. B Biol. Sci.

[b94] Wilson DS, Clark AB, Coleman K, Dearstyne T (1994). Shyness and boldness in humans and other animals. Trends Ecol. Evol.

[b95] Wilson AJ, Réale D, Clements MN, Morrissey MM, Postma E, Walling CA (2010). An ecologist's guide to the animal model. J. Anim. Ecol.

[b96] Wilson AJ, Grimmer A, Rosenthal GG (2013). Causes and consequences of contest outcome: aggressiveness, dominance and growth in the sheepshead swordtail, *Xiphophorus birchmanni*. Behav. Ecol. Sociobiol.

[b97] Winberg S, Schjolden J, Øverli Ø, Pottinger T (2007). Stress and stress coping in fish, behavioural correlates and neuroendocrine mechanisms. Comp. Biochem. Physiol. A Mol. Integr. Physiol.

[b98] Wong SC, Dykstra M, Campbell JM, Earley RL (2008). Measuring water-borne cortisol in convict cichlids (*Amatitlania nigrofasciata*): is the procedure a stressor?. Behaviour.

