# Supplementary material for: How integrated are behavioral and endocrine stress response traits? A repeated measures approach to testing the stress-coping style model
Source: Ecol Evol. 2015 Jan 11;5(3):618–33. doi: 10.1002/ece3.1395 (PMC4328767; doi:10.1002/ece3.1395)
Supplement: Supplementary file 1 [file ece30005-0618-sd1.docx]

SUPPORTING INFORMATION

**How integrated are behavioural and endocrine stress response traits? A repeated measures approach to testing the stress coping style model.**

**Boulton K, Couto E, Grimmer AJ, Earley RL, Canario AVM, Wilson AJ_,_ Walling CA**

Table S1. Estimates of among-individual (V_I_) and residual (V_R_) variance for all traits with standard errors in parentheses. V_I_ can be interpreted as repeatability since (transformed) traits were scaled to standard deviation units. Models were fitted using ASReml and likelihood ratio tests used to determine the statistical significance of V_I_ assuming the test statistic to be asymptotically distributed as a 50:50 mix ofχ^2^_0_ and χ^2^_1_ (following Visscher, 2006). Note however that the assumption of residual normality is violated for emREF, that was analysed here on the observed (i.e. 0/1) data scale. All models contained fixed effects as shown in full in supporting information Table S2.

| **Trait** | **V**_I_ | **V**_R_ | **χ^2^** | **P** |
| --- | --- | --- | --- | --- |
| Track length (TL) | 0.256 (0.137) | 0.737 (0.121) | 8.77 | 0.002 |
| Activity (ACT) | 0.291 (0.146) | 0.708 (0.116) | 11.0 | <0.001 |
| Area covered(AC) | 0.191 (0.120) | 0.792 (0.130) | 5.26 | 0.011 |
| Time in middle (TIM) | 0.054 (0.086) | 0.914 (0.151) | 0.485 | 0.243 |
| Time out of refuge (TOR) | 0.229 (0.129) | 0.750 (0.123) | 7.34 | 0.004 |
| Emergence from refuge (EmREF) | 0.140 (0.103) | 0.736 (0.160) | 3.07 | 0.040 |
| Pre-trial cortisol (F_PRE_) | 0.078 (0.071) | 0.604 (0.099) | 1.89 | 0.085 |
| Pre-trial 11-ketotestosterone (11KT_PRE_) | 0.200 (0.112) | 0.590 (0.098) | 7.62 | 0.003 |
| Post-trial cortisol (F_POST_) | 0.007 (0.074) | 0.930 (0.153) | 0.01 | 0.461 |
| Post trial 11-ketotestosterone (11KT_POST_) | 0.000 (-)* | 0.803 (0.120) | 0.00 | 0.500 |

*With V_I_ constrained to positive parameter space the estimate was bound to zero such that no SE can be estimated.

Table S2. Estimated fixed effects from univariate mixed models of all traits. Models were fitted using ASReml including individual identity as a random effect (see Table S1 for variance component estimates). Conditional *F*-tests were used to assess significance of all fixed effects. Trial number (*Trial*) was fitted as a five level factor. The contrasts among factor levels are not shown here but are depicted in Supporting Information Figure S1. *Day order* was fitted as a linear effect while *Stack* was a two level factor (Effect size indicates the difference for Stack 2 relative to Stack 1). A linear effect of fish *mass* was also included in models of endocrine traits.

| **Trait** | **Fixed effect** | **Effect size (SE)** | **DF** | ***F*** | **P** |
| --- | --- | --- | --- | --- | --- |
| TL | *Mean* | 1.433 (0.265) | 1,18 | 71.8 | <0.001 |
|  | *Trial* |  | 4,74.2 | 1.87 | 0.136 |
|  | *Day order* | 0.021 (0.016) | 1,75.7 | 1.72 | 0.175 |
|  | *Stack* | 0.180 (0.287) | 1,18.7 | 0.390 | 0.539 |
| ACT | *Mean* | 1.265 (0.268) | 1,18.1 | 61.5 | <0.001 |
|  | *Trial* |  | 4,74.2 | 1.50 | 0.228 |
|  | *Day order* | 0.022 (0.015) | 1,75.6 | 1.76 | 0.163 |
|  | *Stack* | 0.265 (0.297) | 1,18.7 | 0.800 | 0.383 |
| AC | *Mean* | 1.610 (0.259) | 1,18 | 107.2 | <0.001 |
|  | *Trial* |  | 4,74.3 | 1.66 | 0.182 |
|  | *Day order* | 0.019 (0.016) | 1,76 | 1.10 | 0.240 |
|  | *Stack* | 0.298 (0.268) | 1,18.8 | 1.24 | 0.279 |
| TIM | *Mean* | 0.895 (0.247) | 1,16.4 | 58.1 | <0.001 |
|  | *Trial* |  | 1,73.1 | 0.830 | 0.513 |
|  | *Day order* | 0.023 (0.017) | 1,76.4 | 1.86 | 0.179 |
|  | *Stack* | 0.459 (0.222) | 1,17.7 | 4.28 | 0.052 |
| TOR | *Mean* | 1.220 (0.261) | 1,18 | 65.5 | <0.001 |
|  | *Trial* |  | 4,74.2 | 1.39 | 0.247 |
|  | *Day order* | 0.028 (0.016) | 1,75.5 | 3.26 | 0.077 |
|  | *Stack* | 0.329 (0.278) | 1,18.6 | 1.40 | 0.251 |
| emREF | *Mean* | 0.853 (0.428) | 1,17.4 | 28.0 | <0.001 |
|  | *Trial* |  | 4,71.3 | 4.42 | 0.003 |
|  | *Day order* | -0.013 (0.016) | 1,73.0 | 0.670 | 0.420 |
|  | *Stack* | -0.513 (0.244) | 1,17.9 | 4.42 | 0.050 |
| F_PRE_ | *Mean* | 6.930 (0.216) | 1,17.1 | 4215.2 | <0.001 |
|  | *Trial* |  | 4,74.7 | 3.27 | 0.012 |
|  | *Day order* | -0.023 (0.014) | 1,76 | 0.67 | 0.106 |
|  | *Stack* | -1.090 (0.216) | 1,18.9 | 29.67 | <0.001 |
|  | *Mass* | -0.039 (0.332) | 1,27.6 | 0.01 | 0.908 |
| 11KT_PRE_ | *Mean* | 13.21 (0.243) | 1,17 | 9828.7 | <0.001 |
|  | *Trial* |  | 4,74.4 | 7.18 | <0.001 |
|  | *Day order* | -0.056 (0.014) | 1,74.8 | 16.11 | <0.001 |
|  | *Stack* | -0.390 (0.270) | 1,18.7 | 1.25 | 0.164 |
|  | *Mass* | 0.466 (0.393) | 1,34.8 | 1.40 | 0.245 |
| F_POST_ | *Mean* | 1.560 (0.244) | 1,91 | 339.6 | <0.001 |
|  | *Trial* |  | 4,91 | 1.27 | 0.339 |
|  | *Day order* | -0.024 (0.017) | 1,91 | 0.44 | 0.966 |
|  | *Stack* | -0.465 (0.216) | 1,91 | 8.52 | 0.020 |
|  | *Mass* | 0.349 (0.342) | 1,91 | 0.93 | 0.339 |
| 11KT_POST_ | *Mean* | 9.822 (0.226) | 1,16.7 | 695.8 | <0.001 |
|  | *Trial* |  | 4,73.2 | 2.61 | 0.022 |
|  | *Day order* | -0.037 (0.016) | 1,77.9 | 22.72 | <0.001 |
|  | *Stack* | -0.368 (0.201) | 1,19 | 0.02 | 0.889 |
|  | *Mass* | 0.003 (0.314) | 1,22.4 | 0.50 | 0.486 |

Table S3. Eigen vector decomposition of the **I** matrix estimated among behavioural traits observed in the modified open field trial prior to the simulated predator attack.

| Eigen Vector | 1 | 2 | 3 | 4 | 5 |
| --- | --- | --- | --- | --- | --- |
| Eigen Value | 1.11 | 0.029 | 0.013 | 0.002 | 0.000 |
| Percentage of variance explained | 96.2 | 2.52 | 1.12 | 0.167 | 0.009 |
| Trait loadings |  |  |  |  |  |
| Track length | 0.491 | -0.444 | 0.198 | 0.513 | 0.510 |
| Activity | 0.523 | 0.058 | 0.325 | 0.184 | -0.764 |
| Area covered | 0.430 | -0.419 | -0.689 | -0.386 | -0.123 |
| Time in middle | 0.274 | 0.697 | -0.505 | 0.412 | 0.125 |
| Time out of refuge | 0.474 | 0.373 | 0.354 | -0.620 | 0.355 |

Figure S1. Estimated effects of trial number (*Trial*) from univariate models of a) behavioural and b) endocrine traits (see Supporting Information Tables S1 and S2 for full results). *Trial* was fitted as a multilevel factor and effect sizes are shown (in standard deviation units) relative to the predicted mean at trial 1. Error bars denote ± SE. There is a general pattern of decrease with trial number across behavioural traits, though *Trial* was only statistically significant for emergence from refuge (Supporting Information Table S2), a result driven by notably higher emergence rates in Trial 3. Significant mean differences among trials were found for all endocrine traits except F_POST_ (Supporting Information Table S2).

**A**

**B**

Figure S2. Posterior distribution of the intraclass correlation (IC) of the binary trait, emergence from refuge (emREF) from an analysis modelling emREF as a categorical trait in MCMCglmm. See main text for full model details.
